# When genomics companies act as biobanks: governance challenges in an era of genomic assetization

**DOI:** 10.3389/fgene.2026.1778236

**Published:** 2026-03-12

**Authors:** Alexis Walker

**Affiliations:** Department of Medical Humanities and Ethics, Columbia University, New York, NY, United States

**Keywords:** bioeconomy, commercial genomics, commercialization, data governance, ownership, political economy, privacy, venture capital

## Abstract

**Introduction:**

Contemporary genomics companies increasingly perform core biobanking functions—collecting, storing, and sharing large volumes of genetic data and biospecimens—without being widely recognized or governed as biobanks. At the same time, these holdings are increasingly undergoing assetization: the treatment of genomic data and biospecimens as corporate assets subject to sale, acquisition, and valuation as indicators of company worth. This paper examines governance challenges arising from this transformation, particularly when companies experience restructuring, acquisition, or insolvency.

**Methods:**

Using a comparative qualitative case study approach, the paper analyzes three examples: a Nigerian genomics company created to expand African representation in genomics, DNA marketplaces that aggregate and license genetic data, and recent events surrounding the attempted sale of 23andMe’s genomic holdings. The analysis draws on scholarship on biobanking governance, commercialization in biomedicine, and justice-oriented critiques of genomic research.

**Results:**

The paper identifies governance issues that have emerged as biobanking technologies and organizational forms have evolved. Commercial genomics infrastructures expose gaps in existing biobanking governance, regarding the way data and samples are stewarded during organizational change, oversight of financial pressures in custodial decision-making, participation incentivizes, and public understandings of biobanking that are shaped by consumer and other commercial genomics infrastructures.

**Discussion:**

These findings highlight the need for governance approaches that better reflect todays interconnected biobanking ecosystem, which includes public and nonprofit biobanks, government-academic-industry collaborations, and companies operating as *de facto* biobanks. Strengthening governance may require clearer planning for closure and transition, including limits on the transfer or sale of data and biospecimens during restructuring, possible roles for public stewardship, and a more explicit role for established biobanking institutions in developing shared governance standards across the genomics ecosystem.

## 1 Introduction

Genomics companies have amassed some of the world’s largest repositories of human genetic data and biospecimens, and their organizational models are often oriented toward making these available for research by third parties—typically through arrangements involving financial or other forms of value exchange with industry or academic researchers (Sturdy, 2025; Spector-Bagdady, 2025). Organizations that operate this way include not only direct-to-consumer (DTC) genetic testing services like 23andMe, but a growing range of companies whose business models depend on aggregating and brokering access to genetic and health data and samples—from genetic data-diversification firms like 54gene to DNA marketplaces that pay consumers for research use of their genetic data, like LunaDNA (Adetiba and Walker, 2025; Walker, 2025b; Howard et al., 2015; Geiger and Gross, 2021). Although these companies occupy core biobanking functions of collecting, storing, and circulating genetic materials for research, they are typically governed through frameworks designed for consumer transactions and commercial data services. In other words, they function as biobanks in practice, while remaining outside the governance expectations and normative assumptions broadly associated with biobanking—such as long-term stewardship and public-interest obligations.

The work of these companies shapes how members of the public understand genetic research and biobanking, without being subject to the expectations, norms and practices of the biobanking arena (Boyce et al., 2019; Nicol et al., 2016; Zawati et al., 2011). Failing to include such genomics companies in discussions of biobanking constrains dialogue across relevant literatures—on biobanking, commercial genomics, and data governance—leaving key conceptual tools, empirical findings, and governance lessons underutilized. Central to this shift is the growing *assetization* of genetic resources: the treatment of genomic data and biospecimens as corporate assets subject to sale, acquisition, and valuation as indicators of company worth—especially at times of organizational restructuring or insolvency (Birch and Muniesa, 2020).

This paper highlights governance lessons for biobanking systems by examining genomics companies that broker access to genetic data and biospecimens as *de facto* biobanks—what could be called “companies-as-biobanks.” By “companies-as-biobanks,” I refer to genomics firms that publicly operate as consumer genomics or health technology startups, but whose business models also depend on building, maintaining, and monetizing large-scale repositories of genetic data and biospecimens for third-party research use. This is distinct from “commercial biobanks,” which operate more obviously as biorepositories, with biobanking functions primary to their public corporate positioning (Samuel et al., 2022). Commercial biobanks typically collect samples and consent patients in clinical or hospital contexts, framing their sample and data collection as contributing to the greater good by making these resources available for further health research—although the for-profit nature of their work is often not especially visible to contributors (Josefson, 2000). This structure differs markedly from the growing group of companies that frame themselves as consumer genomics or health technology firms, while at the same time performing significant biobanking functions that they rarely foreground in their public relations.

These latter companies-as-biobanks that are the focus of this paper typically adopt Silicon Valley–style identities as technology startups, complete with venture capital financing, platform logics, and rapid scaling imperatives. Many rely on two-sided business models that foreground consumer-facing services—such as ancestry reports or health insights—while rendering the brokering of genetic data and biospecimens to third parties less visible to participants (Anderlik, 2003; Stoeklé et al., 2016). In some cases, however, particularly in DNA marketplace models, the sale or licensing of genetic data is made an explicit and central feature of participation, directly linking contribution to downstream value creation (Ahmed and Shabani, 2019). Drawing on a set of cases in commercial genomics globally, this paper shows how such companies both fit within and unsettle contemporary biobanking arrangements, particularly with respect to continuity of governance, protections for contributors, and patterns of participation across biobanking settings.

The paper uses a combination of justice theories to anchor its case analysis and governance recommendations. This includes egalitarian accounts emphasizing fairness in access to healthcare and the distribution of research benefits, as well as justice frameworks that have focused on common ownership of biological resources and ethical limits on commodification (Lebacqz and Gaudet, 2025; Ekmekci and Arda, 2015; Rawls, 1971; 1958; Dworkin, 1981; Christensen, 2009; Wilson, 2004). The analysis also centers critiques of a growing reliance on informed consent as the primary basis of participant protection—and the problems of such outsourcing of protection, especially given consistent findings that many participants have limited understanding of the studies they consent to, even under robust consent processes (Sutter et al., 2020; Xu et al., 2020; O’ Sullivan et al., 2021; Eisenhauer et al., 2019; Beskow and Weinfurt, 2019; Kasperbauer et al., 2021; Barn, 2025; Solberg and Steinsbekk, 2015). Instead, the paper argues that durable justice obligations to participants depend on institutional arrangements and regulatory structures that protect participants well beyond consent, and that can withstand shifting organizational and technical infrastructures of biobanking.

Biobanking spans many specimen types and associated data infrastructures, from disease-focused collections to population-scale repositories. This paper focuses on genomic settings, including both biospecimens and the genetic datasets derived from them. The governance tensions examined here are especially visible in relation to genetic data infrastructures, as genomic data are durable, digitally transferable, scalable across populations, and reusable across multiple research domains. These characteristics make them particularly compatible with platform-based business models, cross-sector data aggregation, and contemporary company valuation practices. Questions of sample custody, transfer, and disposition remain integral to the analysis—especially during restructuring or closure—while recognizing that assetization dynamics tend to crystallize most clearly around genomic data.

The paper proceeds in three steps. First, it situates genomics companies within the broader biobanking landscape by tracing shifts in biobanking technologies and organizational forms, including the expansion of large, public and non-profit population-based and networked infrastructures that are now aging, financially strained, and, in some cases, facing closure. Second, it examines cases from three segments of the commercial genomics industry—direct-to-consumer testing, data-diversification firms, and DNA marketplaces—to illustrate how financial pressures, venture-backed valuation logics, and institutional transition within commercial genomics shed light on governance challenges across the wider biobanking ecosystem, including questions of incidentality and intentionality. Third, it considers the governance implications of these developments, focusing on lessons from these cases that can support clearer planning for continuity, stewardship, and participant protection across biobanking settings. Together, the analysis aims to align biobanking governance with contemporary genomic data economies by integrating insights from biobanking, commercial genomics, and data governance to support more realistic, durable, and protective approaches to stewardship across the biobanking landscape.

### 1.1 The evolving landscape of biobanking

This section examines how, over the past three decades, shifts in biobanking technologies and institutional forms—including digitization, networked infrastructures, and the emergence of companies-as-biobanks—have transformed the way biological materials and data are collected, stored, and shared. These changes have brought new challenges to the foreground, particularly around continuity of stewardship, responsibility across organizational change, and the protection of participants when biobanking infrastructures evolve, fragment, or dissolve. Policy frameworks developed for relatively stable, institutionally bounded biobanks have struggled to keep pace with these dynamics. Understanding these challenges requires closer attention to how biobanks have evolved in practice: how their technical infrastructures, organizational arrangements, and normative expectations have changed over time, and how those changes reshape the ethical and regulatory questions biobanking now raises.

#### 1.1.1 From study repositories to large population biobanks

Although researchers have long collected biological specimens, the term *biobank* gained traction in the 1990s as studies began assembling ever larger numbers of samples—supported by automation, web-based infrastructures, and increasing computational capacity (De Souza and Greenspan, 2013). Early examples were often small, study-specific, or hospital-based collections built to support disease-focused research. But biobanks grew in the 1990s alongside the HIV/AIDS research enterprise; many repositories emerged under urgent conditions of this epidemic and relied heavily on participant advocacy, community governance, and reciprocal relationships between researchers and patient or at-risk groups (De Souza and Greenspan, 2013; Walker et al., 2021). These origins helped establish expectations that repositories of biological materials be embedded in institutions oriented toward protection, accountability, and collective benefit.

Advances in robotics, digital recordkeeping, and high-throughput genotyping helped facilitate the expansion from study-specific collections to large population-scale biobanks. While earlier repositories sometimes linked samples to clinical information or longitudinal follow-up, late-1990s and early-2000s initiatives in Iceland, the United Kingdom, Estonia, and elsewhere institutionalized these practices at national scale, systematically linking biospecimens with health records and ongoing life-course data. This transformation positioned biobanks as durable research infrastructures rather than finite study resources (Coppola et al., 2019). Iceland’s collaboration with deCODE Genetics is an early and influential example of how these developments intersected with public–private partnership models. In this late 1990s case, the Icelandic government funded and led the collection of a population-wide biobank, and subsequently granted a private company exclusive access to the resource—foregrounding ethical tensions around consent, governance, and public benefit that would recur as biobanking scaled globally (Fortun, 2008).

As biorepositories expanded in scale and scope, debates also emerged over how to name and conceptualize these new research infrastructures. In the early 1990s in the US, biospecimen collections for research were often called “biorepositories; ” today this definition has continued to be applied to biobanking (Watson et al., 2019). In 1999, when an international society was founded to address issues in biological sample management, they adopted the term “repository”: the International Society for Biological and Environmental Repositories. However, many scholars and practitioners have argued that “bank” and “repository” language over-emphasize *storage*, contributing to a focus on collection rather than distribution, access and sharing; some observers have advocated for versions like “biotrust,” or other terms that might better reveal the diverse purposes and motivations for various kinds of collections—be that a focus on custodianship, research support, commercial profit (Watson et al., 2019). Daniel Catchpoole, for example, has argued that the term “biobank” obscures these various purposes and thus impedes the setting of appropriate expectations and ethical boundaries for specific repository types (Watson et al., 2019). These naming debates continue to capture anxieties about purpose, responsibility, and the ethical limits of collecting large volumes of biological material under evolving scientific and commercial conditions.

Governance frameworks for biobanking have since been consolidated around norms for ethics review, transparency, community engagement, and long-term stewardship (Yaghoobi and Hosseini, 2023). These norms have contributed to public representations of biobanks as stable, publicly oriented infrastructures, even as commercial actors have remained integrally intertwined in biobanking practices—through partnerships, licensing arrangements, service provision, and commercially-owned banks (Anderlik, 2003). Alongside public and academic biobanks, in the early 2000s a parallel ecosystem of commercial biobanking firms—such as Ardais, Asterand, and Genomics Collaborative—emerged to collect, bank, and distribute human biospecimens through contractual agreements with hospitals and clinical sites, often supplying samples directly to downstream research or industry users rather than maintaining long-term public repositories (Somiari and Somiari, 2015; Anderlik, 2003; Josefson, 2000). These models further blurred distinctions between stewardship-oriented biobanking and market-oriented specimen circulation. And yet, research on public trust in biobanks has consistently shown greater confidence when repositories are based in academic and non-profit institutions, with concerns about commercialization and data sharing shaping public willingness to participate (Sanderson et al., 2017; Tozzo and Caenazzo, 2020; Hall et al., 2010; Brall et al., 2021).

Global research on biobank participation has shown that individuals’ decisions to contribute biological materials are shaped by a combination of motivations, including contribution to the common good, expectations of personal or familial benefit, trust in institutions, and beliefs about how data will be governed over time (Critchley et al., 2015; 2012; McDonald et al., 2014; Halverson and Ross, 2012; Amin et al., 2018). As biobanking infrastructures have evolved, fulfilling these expectations has become increasingly complex. Growth in scale, scope, and interconnection has meant that stewardship commitments must now operate across infrastructures that extend beyond single institutions or bounded repositories, introducing new tensions around responsibility, continuity, and oversight. These developments set the stage for the governance challenges associated with digitization, data mobility, and distributed biobanking systems examined in the following section.

#### 1.1.2 From physical repositories to distributed hybrid systems

Biorepositories of the 1980s and 1990s were tightly coupled to physical biospecimens and local institutional infrastructures. But coordinated investments across genomics, informatics, and population-scale research have driven a shift in biobanking toward larger, data-intensive networks and nodes (Brancato et al., 2024). As collaborations have multiplied across universities, hospitals, national research systems, and international consortia since the late 1990s, digitalization has helped further decouple biobanking governance concerns from physical specimens alone. Many risks associated with biobanking—such as secondary use without robust oversight, unclear custodianship obligations, and long-term governance vulnerabilities—are now features of genomic data ecosystems more broadly, not just of institutions that maintain physical biospecimens.

Consequently, some scholars and governance bodies have begun to treat biobanks and health databases as a shared category for analysis and governance (Walshe et al., 2024; Amoakoh-Coleman et al., 2023; Caenazzo et al., 2015)—sometimes referred to as “data-banking” (Stoeklé et al., 2016). There are, of course, distinct ethical issues that arise with the management of physical samples, and across biobanks with various national contexts, research practices, and structures—including population-specific, disease-specific, and commercial settings (Aicardi et al., 2016). But as digital biobanks of imaging and other health data have proliferated, and genomics research increasingly focuses on secondary data analysis, the infrastructures and functions of genetic databases and sample repositories increasingly overlap.

The management of large-scale genetic datasets for third party use—whether tied to physical samples or operating as digital biobanks—raises consistent biobanking concerns around consent durability, expectations of stewardship, transparency of data access, benefit-sharing, and so on (Zawati et al., 2011). As genomic infrastructures have become more distributed, data-intensive, and digitally mobile, the activities that constitute “biobanking” increasingly occur well beyond the institutional contexts where the term originated—including in the context of genomics companies, platforms, and data brokers (Wolf et al., 2020). This expansion has not only blurred institutional boundaries, but also intensified questions about what happens to biobanking commitments as infrastructures age, change, or come under strain—as discussed in the following section.

#### 1.1.3 Aging biobanks

Early population biobanks and large research repositories were often built with optimism about future scientific demand, yet many have since confronted persistent problems of high operating costs, underutilization of stored specimens, and uncertain sustainability (Stephens and Dimond, 2015). Operational models for maintaining physical samples, associated data systems, and regulatory compliance over decades have required far more external funding than initially anticipated, a pressing challenge as funding priorities shift and research practices evolve (Tupasela and Stephens, 2013). Biobanks have consistently suffered from low rates of sample access and secondary use, raising ethical questions about whether participants’ contributions are being meaningfully honored, as well as practical difficulties with cost recovery (Rao et al., 2019).

In response to these pressures, many public and nonprofit biobanks have increasingly turned to commercial partnerships as a means of sustaining operations, supporting data infrastructure, or enabling downstream research use. While such arrangements can extend the scientific life of collections, they also introduce new dependencies and governance risks, particularly when long-term stewardship relies on private actors whose commitments are shaped by shifting market and organizational priorities (Caulfield et al., 2014).

A number of public, nonprofit, and commercial biobanks have shut down in recent years due to financial pressures, often through closeout processes that raise difficult questions about consent, stewardship obligations, and the permissibility of treating biological materials as assets once a biobank ceases operation (Baláž et al., 2022). For example, in the summer of 2025, the California Institute for Regenerative Medicine (CIRM) announced the closure of its human stem cell biobank, which had been established through a voter initiative and was once the largest stem cell repository in the world (Wosen, 2025).

Despite receiving tens of millions of dollars of state funding, the CIRM biobank faced challenges familiar across the biobanking landscape, including lower than anticipated usage, outweighing annual maintenance costs. These pressures became acute when the private company responsible for storing samples and fulfilling orders—an arrangement initially provided at no cost as part of a broader collaboration around induced pluripotent stem cell production—announced that it would no longer manage the repository without compensation. During closing, CIRM offered thousands of cell lines for rapid sale at steeply discounted prices in order to move the samples (Wosen, 2025). While CIRM retained archival samples of each line, the story illustrates how aging biobanks—even those founded on explicit public-interest mandates—can come to treat biological materials as liquid assets, raising questions about stewardship, access, and public accountability as infrastructures age. This case also illustrates how public biobanking infrastructures can become vulnerable when long-term stewardship depends on private partners whose commitments are contingent and time-limited—an increasingly common arrangement across genomics (Granados Moreno et al., 2017; Senier et al., 2015; Walker, 2025a).

These challenges are compounded by a widespread lack of advance planning for institutional decline in biobanking. A recent survey found that 74% of public and non-profit biobanks have no written plans for what will happen in the case of biobank closure, and do not address the issue in their consent forms (Rao et al., 2019). The absence of legacy planning leaves unresolved questions about custodianship, participant expectations, and the ethical status of samples and data once a biobank can no longer operate as intended–issues that are particularly acute in the rapid turnover environment of the genomics startup world, and that increasingly confront biobanking infrastructures more broadly.

### 1.2 Commercial genomics companies as biobank actors in the biotrust ecosystem

Biobanking today takes place across a range of institutional settings, including public and nonprofit repositories, population biobanks with structured industry access (such as UK Biobank and FinnGen), disease-specific academic biobanks supported through translational partnerships, and genomics companies that manage genetic data and samples at scale. These arrangements are increasingly interconnected through shared research practices and overlapping participant populations; nearly a quarter of the US population has participated in consumer genetic testing alone (Makhnoon et al., 2024). Although growth in direct-to-consumer genetic testing has slowed in the United States, uptake is increasing in many other regions, where expansion is often framed as a means of diversifying global genetic databases—an expectation that raises governance issues addressed later in this paper (Kang et al., 2021). Taken together, these developments point to a biobanking ecosystem in which different actors engage in overlapping practices of genetic and clinical data and sample storage, access governance, and third-party sharing, even as institutional mandates and incentives diverge.

Members of the public encounter genomics through multiple entry points across this ecosystem, including clinical research studies, population biobanks, direct-to-consumer testing services, and data marketplaces. While participants may recognize distinctions among these settings, empirical research suggests that experiences in one domain can inform expectations in others, particularly with respect to how genetic data are handled, protected, and reused (Boyce et al., 2019; Briscoe et al., 2020; Walshe et al., 2024). From this perspective, the health of biobanking ecosystems depends not only on the governance of individual repositories, but also on how different biobanking actors collectively shape contributor protections as well as public understandings of participation. Recognizing these interdependencies provides a foundation for examining governance challenges that cut across both traditional and commercial biobanking contexts—such as legacy planning, incentives for participation, and the treatment of genetic data as assets.

Against this backdrop, many genomics companies now curate large-scale genetic datasets, manage participant interfaces, facilitate secondary use, and broker access for research or commercial partners. Despite these biobanking functions, such companies strategically frame themselves—and are regulated—as consumer services, technology companies, or research-support vendors. This positioning has precedent: the DTC genetics industry has a history of classifying its offerings in ways that secure lighter regulatory oversight, for example, by framing them as entertainment tools to avoid FDA oversight, even when participants perceive them as health-related (Jabloner, 2019; Parthasarathy, 2014). Importantly, research on more traditional biobanks has also shown that institutional actors engage in strategic framing to manage tensions around access, value creation, and economic extraction (Baláž et al., 2022bib_baláž_et_al_2022b). Public-facing communications typically emphasize scientific advancement, solidarity, and public benefit, while economic arrangements—including revenue-generating access fees and agreements, intellectual property management, and partnerships with commercial actors—are framed as necessary infrastructure rather than as central features of the biobank’s value model (Corrigan and Tutton, 2009). In this way, political-economic dimensions of are backgrounded or narratively subordinated to public-good narratives (Mitchell, 2012).

Recognizing genomics companies as part of the biobanking ecosystem brings their influence on public understandings of both genetic data stewardship and the custody of underlying biospecimens into clearer view. It also exposes a structural mismatch between traditional biobank expectations—stability, accountability, and long-term responsibility—and the operational realities of today’s biobanking landscape, which are increasingly shaped by commercial logics. Situating commercial genomics companies within biobanking debates therefore serves two analytic purposes. First, the pace and volatility of commercial genomics bring into sharper relief governance challenges that are also emerging, more gradually, across more traditional biobanking, including questions of sustainability and continuity. Second, it underscores the importance of involving traditional biobank institutions, policymakers, and international research organizations in shaping governance mechanisms that include companies-as-biobanks, whose operations increasingly influence public trust and participation in biobanking more broadly. The following section presents a series of case studies that highlight these issues.

## 2 Materials and methods

### 2.1 Study design

This study uses a qualitative, comparative case study approach to examine how genomics companies increasingly perform core biobanking functions, and the governance challenges that arise as a result. Case studies were selected to capture variation across organizational models, geographic contexts, and modes of participant engagement, while illuminating shared structural dynamics relevant to contemporary biobanking governance.

The selected cases are not presented as isolated scandals or exceptional failures, but as sites where structural tensions inherent in contemporary genomics business models became publicly visible. Each case represents a distinct organizational configuration: a venture-capital–financed genomics company focused on expanding African representation in global datasets (54gene); a consumer-facing genomics platform whose data holdings became central during bankruptcy proceedings (23andMe); and DNA marketplace models that explicitly link participation to data monetization. While not all genomics companies experience public dispute or insolvency, the financial and corporate logics at issue—venture capital financing, valuation tied to data holdings, platform-based revenue models, and cross-border investor relationships—are widespread within the sector. These cases therefore illuminate governance vulnerabilities that may arise across diverse commercial genomics enterprises globally.

Although the case analysis centers on Nigeria and the United States, the governance dynamics examined arise from financial and institutional logics—particularly venture capital financing, cross-border investment, platform-based data markets, and valuation tied to proprietary data holdings—that increasingly structure genomics enterprises worldwide. These dynamics are not unique to Nigeria or sub-Saharan Africa; technology hubs such as Lagos have become part of a broader global landscape of venture-backed innovation ecosystems (Jiménez and Zheng, 2018; Fatema and Raza, 2024; Atiase et al., 2020). At the same time, cross-border investment can amplify governance vulnerabilities when corporate actors operate across jurisdictions with uneven regulatory authority, fragmented oversight, or limited coordination between financial and research governance regimes. The Nigerian case thus illustrates how transnational capital relationships—and the possibility of regulatory arbitrage—shape biobanking governance across jurisdictions, rather than reflecting deficiencies specific to any one setting.

### 2.2 Data sources

The analysis draws on publicly available, narrative data sources, including:

- Corporate filings, investor disclosures, and regulatory documents
- Publicly available company policies, consent materials, and participant-facing communications
- Legal filings, court records, and legislative materials related to data protection and bankruptcy
- Media reporting, investigative journalism, and industry analyses

These materials were collected 2017–2025, and analyzed to trace organizational change, governance practices, and evolving public and regulatory responses. Media reporting and industry analyses were used both to reconstruct timelines of events and to examine how governance controversies were publicly framed. Because public trust and participant expectations are central to biobanking governance, attention was paid to how cases were represented in widely circulated accounts and policy commentary. Where possible, factual claims drawn from journalistic sources were cross-referenced with primary materials such as corporate filings, court records, legislative documents, and official company communications. Media sources were not treated as definitive evidence of disputed facts, but as part of the broader ecosystem through which governance practices and public understandings are shaped.

Materials were identified through sustained monitoring of public reporting, industry discourse, legal developments, and regulatory debates related to commercial genomics and biobanking between 2017 and 2025. This monitoring occurred within the context of an NHGRI-funded study examining ethics and governance in private-sector genomics.

### 2.3 Analytic approach

Materials were analyzed using qualitative document analysis and comparative thematic analysis. Attention was paid to governance dimensions that recurred across cases as sites of public dispute and legal uncertainty. The analysis was informed by established justice frameworks in bioethics and political philosophy, as well as legal scholarship on data governance, consent, and custodianship, which were used as interpretive lenses rather than as prescriptive evaluative tools.

All data analyzed were publicly available or drawn from published sources. No human subjects research was conducted, and no identifiable personal data beyond what has been publicly reported were collected or analyzed.

### 2.4 Justice frameworks used for case analysis

Debates about governance in biobanking are ultimately debates about justice: who bears the burdens of research participation, who benefits from scientific and commercial advances, and how responsibilities are distributed across institutions that manage biological materials and data. The Ethics Committee of the Human Genome Organization (HUGO) has highlighted three distinct but overlapping dimensions of justice as especially relevant to genomics: compensatory justice—concerning recognition or recompense for contribution; procedural justice—concerning inclusive and accountable decision-making processes; and distributive justice—concerning equitable access to healthcare, research benefits, and scientific resources (Knoppers et al., 2000). These dimensions often come into tension. For example, commercial activity may accelerate research translation, but it may also restrict access through pricing, licensing, or data control mechanisms that exclude populations most in need of resulting interventions (McCoy, 2025).

Philosophical theories of justice help clarify what is at stake for biobanking governance. Egalitarian approaches, including Rawlsian justice as fairness and Dworkinian equality of resources, focus on ensuring that social arrangements do not leave some individuals systematically worse off than others—whether assessed against a common standard or in relation to an individual’s own judgments about what matters for their life (Rawls, 1971; 1958; Dworkin, 1981). Applied to biobanking, these frameworks invite scrutiny of whether research infrastructures are designed in ways that predictably disadvantage some groups, limit their access to downstream benefits, or expose them to greater risk over time than others (Critchley et al., 2016).

Other justice-oriented frameworks foreground questions of ownership, control, and commodification. From common-ownership and related perspectives, biological materials and data derived from human bodies raise distinctive justice concerns because they are simultaneously individual, relational, and collective in character (Gümplová, 2023; García-López et al., 2021; Garba, 2018; Gold, 1996; Parry, 2008; Cahill, 2001). Treating such materials as tradable assets may help incentivize innovation, but it also raises questions about who has legitimate claims over use of biological resources, who bears the risks of their circulation, and whether market-based governance adequately reflects shared interests in health, knowledge, and social welfare. Here, justice issues arise not only in terms of commercialization *per se*, but also when commodification reallocates power and value in ways that limit the public’s ability to contest or influence decisions.

Reparative justice perspectives further expand the scope of analysis by situating biobanking within broader histories of social and economic extraction. These approaches emphasize that marginalized populations have contributed not only to scientific advancement but to societal wealth more broadly, while often being excluded from its benefits (Harman et al., 2021; Agozino, 2021; Danieli, 2009; Soled et al., 2021). In this view, the ethical stakes of genomics research extend beyond fair research practice to questions of how institutions respond to durable inequalities in healthcare access, political voice, and material security. Reparative frameworks therefore shift attention from speculative future benefits toward present-day obligations to address structural disadvantage through governance, benefit-sharing, and institutional accountability.

Across these perspectives, a shared concern is that justice cannot be secured through consent or market participation alone (Barn, 2025; Hall et al., 2010; Sutter et al., 2020; Xu et al., 2020; Eisenhauer et al., 2019). Instead, justice-oriented biobanking governance requires institutional arrangements that ensure durable protections, fair benefit distribution, and accountability over time—especially in systems where biological materials generate both scientific knowledge and economic value. These frameworks provide a foundation for evaluating the cases in this paper and for assessing whether contemporary biobanking practices align with broader commitments to equity, reciprocity, and the common good.

### 2.5 Study limitations

This study relies on publicly available materials, including corporate disclosures, legal filings, policy documents, and media reporting. As such, the analysis captures governance dynamics at the point they become publicly visible, rather than internal deliberations or confidential negotiations that may shape institutional decision-making. While media accounts were triangulated with primary documents where possible, reporting biases and incomplete information remain possible.

The case selection is analytically purposive rather than systematic. This study does not present a comprehensive survey of all commercial genomics firms, nor a coded review of media coverage. Instead, it draws on an informed selection of cases in which assetization, custodial transition, and participant-protection tensions became sufficiently visible to generate public documentation. It is likely that additional cases involving similar dynamics have occurred but did not receive sustained international reporting and therefore remain less accessible to analysis. The absence of systematic enumeration limits claims about prevalence. At the same time, the existence of multiple publicly documented cases across distinct jurisdictions suggests that the governance dynamics identified here are not isolated anomalies.

The analysis also draws primarily on English-language materials, which may constrain access to jurisdiction-specific debates in non-Anglophone contexts. Finally, the governance recommendations offered are normative and forward-looking; their feasibility will depend on legal, political, and institutional conditions that vary across settings.

## 3 Results

Case studies in commercial genomics and biobanking governance. To investigate how genomics companies fit within and illuminate key governance challenges in today’s biobanking ecosystem, this section turns to a set of case studies from commercial genomics. These cases illustrate how companies that collect, store, and circulate genetic data and biospecimens operate within biobanking systems, exposing pressures around financing, valuation, continuity, and participant protection that existing frameworks struggle to address. The section begins with 54gene, a Nigeria-US-based genomics company whose focus on African genetic data makes visible the ways that efforts to correct scientific underrepresentation intersect with new forms of assetization and institutional precarity. As noted above, assetization here refers to the process of making items into capitalizable objects capable of being owned and traded (Birch and Muniesa, 2020).

### 3.1 54gene: biobanking assetization and genomic diversity

With the growth of large-scale population biobanks and genome-wide association studies in the early 2000s, population geneticists and others increasingly documented the severe overrepresentation of people of European ancestry in genomics databases and studies internationally (Popejoy and Fullerton, 2016). African populations—who represent the greatest human genetic variation globally—remain dramatically underrepresented, creating not only scientific limitations but also ethical and political concerns: a structural inequity that can constrain the potential of precision medicine and reproduce global disparities in biomedical research (Byiringiro et al., 2025; Corpas et al., 2025; Fatumo et al., 2022; Shaaban and Ji, 2025; Landry et al., 2018; Gurdasani et al., 2015; Sirugo et al., 2019; Tutton, 2009).

In early 2019, within this scientific and moral landscape, a group of internationally-trained Nigerian scientists, engineers, and management professionals launched a startup aimed at building large-scale genomic infrastructure on the African continent, aimed at supporting global precision-medicine initiatives (Idris, 2019). Originally founded as a genomics diagnostics company under the name Stack Dx, the team received seed funding from the influential American venture capital firm and startup accelerator Y Combinator—known for launching companies such as AirBnB, Instacart, and Reddit (Jackson, 2019; Adeo, 2019). After completing Y Combinator’s mentored Silicon Valley residency program, Stack Dx pivoted toward a model centered on building, maintaining, and brokering research access to an African DNA biobank (HealthNews, 2019), and established a US-based holding company (Oladunmade, 2025).

After this pivot, the company’s co-founders—who included researchers and practitioners trained in immunology, biochemistry, medicine, computer science, and business—moved quickly to secure formal agreements with Nigerian hospitals and health systems to recruit patients as biobank participants (Williams, 2020). The company established partnerships with Illumina, a leading global DNA sequencing firm, to build a genomics facility in Lagos, and with Amazon Web Services (AWS) to support cloud-computing and data storage, while also recruiting executives from Genentech and other leading major biotech companies (OddUp, 2021). 54gene also developed partnerships with biopharmaceutical companies seeking to access the growing biobank (OddUp, 2021). In practice, 54gene operated simultaneously as a repository of physical biospecimens, a generator of genomic and phenotypic data, and an intermediary enabling downstream research and development.

54gene raised several rounds of venture and global health funding, ultimately raising more than $45 million from a combination of Silicon Valley venture capital, life-science investment firms, and philanthropically backed global-health funds, including the Gates Foundation (Kazeem, 2022). In this setting, “diversity” took on dual scientific and commercial valences: African genomic data were positioned as essential for improving precision medicine, but also as an asset class capable of generating revenue through partnerships with pharmaceutical companies, analytics firms, and international research consortia. This produces a familiar double bind: communities previously rendered invisible within genomics research became newly visible once their data acquire market value, yet without corresponding guarantees of benefit-sharing and health access (Jabloner and Walker, 2023; Noble, 2013; Bradley, 2025). As discussed in the following section, the assetization of genomic diversity raises governance questions that go beyond commercialization alone. While participation is framed as a way to increase research attention and potential health benefits, it also presents exploitation risks.

Traditional biobanks may face challenges of funding and longevity, but they are typically embedded within frameworks that assume long-term stewardship, oversight continuity, and obligations to participants and publics. By contrast, 54gene’s biobank-like activities unfolded within a venture-capital environment shaped by short investment horizons, competition for market share, and strategic pivots oriented toward commercial viability rather than sustained custodianship. These divergent institutional logics became especially visible when the company began to experience financial problems a few years after its founding.

In 2022 especially, 54gene began to struggle as its founding CEO Abasi Ene-Obong stepped down, alongside several key executives (iAfrikan, 2023). After two additional rapid CEO turnovers, escalating financial strain, and multiple rounds of layoffs, in late 2023 the company began seeking buyers for its valuable repository of genetic samples and clinical data derived from approximately 130,000 participants in West Africa (Ayetoto-Oladehinde, 2023). Numerous reports of the Silicon Valley darling’s rapid rise and fall peppered the tech press, each offering lessons learned from the case (Ayetoto-Oladehinde, 2023; iAfrikan, 2023; Oladunmade, 2025).

In the following years, Ene-Obong alleged that the company’s US and global investors pushed it toward insolvency in order to liquidate its holdings of biospecimens and data for sale: making extreme financial decisions but rejecting a $110 million rescue package, preferring to assetize the valuable specimen bank (Ekhator, 2024). The co-founder’s allegations against investors include using a “supervisory committee” to circumvent the company’s board, moving biological samples from the US-based holding company to its Nigerian subsidiary—with more permissive legal protections, and accepting an initial offer that greatly undervalued the company’s biospecimens in favor of short term gain—a sale later blocked by the Nigerian High Court (Fakiya, 2025). The circumstances surrounding the collapse are now the subject of ongoing legal proceedings, but the Federal High Court in Lagos has granted an injunction preventing the sale of assets pending resolution of the dispute (Oladunmade, 2025). Nigeria’s Data Protection Act of 2023 classifies health and genetic information as sensitive personal data, subject to heightened requirements for lawful processing and cross-border transfer (Aloamaka, 2025; Ekpo et al., 2025). In addition, Nigeria’s National Health Act of 2014 contains provisions restricting the commercialization of human tissue, generally prohibiting the sale of human biological materials except for permitted cost-recovery payments (Iyioha and Nwabueze, 2015; Chukwuma and Obi, 2025; Abonyi and Ejeagu, 2023).

The allegations against 54gene’s global investors expose a fundamental vulnerability: when biospecimens and genetic datasets are treated as corporate assets, there is a strong incentive towards financialization over custodianship—subject to financial restructuring, investor priorities, and the threat of liquidation. For participants and research partners, 54gene’s collapse generated uncertainty about the status and future use of materials contributed under research-oriented consent processes. These dynamics foreshadow similar governance concerns raised by the bankruptcy of a far more established commercial genomics firm—23andMe—which is the focus of the following section.

### 3.2 23andMe: financial stress, legacy planning, and the sale of biobank holdings

The dynamics exposed by the 54gene case are not unique to emerging genomics ventures, resource-constrained settings, or the global South. 23andMe—the most famous and longstanding consumer genomics company in the world—filed for bankruptcy in 2025, raising questions about whether users’ genetic data and samples could be sold along with the company. Importantly, these concerns resonate beyond the commercial sector, echoing challenges increasingly faced by public and nonprofit biobanks as well, where financial strain and closure have likewise prompted difficult questions about the disposition of genetic resources.

23andMe has long positioned itself as a research-driven enterprise, publishing genetic association studies, partnering with academic researchers, and using the language of human subjects research in participant communications (Servick, 2015). These strategies echo the public-facing norms of academic biobanks, and they help produce a sense of alignment with scientific transparency, public good narratives, and stable institutional stewardship (Curnutte and Testa, 2012; Groot et al., 2025; Lehmann et al., 2012). But while participants often experience 23andMe as a health or ancestry service, the company’s business focuses on using participant data to generate revenue by licensing it to pharmaceutical companies and biotechnology firms. These functions are often obscured by the consumer-facing side of the business model, and yet the company is accountable not to public oversight bodies—as in the case of non-profit biobanks—but to investors and shareholders (Stoeklé et al., 2016). These market incentives produce pressures for monetization that sit uneasily with biobanking expectations of stewardship, long-term custodianship, and public accountability, in spite of the elements of 23andMe that operate as a *de facto* biobank—collecting, storing, and providing access to genetic data and samples (Bagdady et al., 2020).

Tensions between the consumer and biobanking sides of 23andMe’s business have become especially visible following a major data breach in late 2023, which exposed the genetic and genealogical information of nearly seven million users (Warner and Andrews, 2025). The scale of the breach drew widespread public attention and prompted an outpouring of user concern about the long-term protection of genetic data in various contexts, alongside policy debates over the adequacy of consumer-privacy frameworks for biobank-scale datasets (Kwon, 2025). In September 2024, 23andMe reached a $30 million settlement with users affected by the breach, who had brought a class-action lawsuit (Walrath-Holdridge, 2024). The financial and reputational fallout from the breach played a significant role in the company’s subsequent bankruptcy filing the following year (Branch, 2025).

The 2025 bankruptcy proceedings crystallized public concern about whether genetic samples and data could be treated as salable assets, and whether the consent agreements under which they were collected meaningfully constrained such transfers (Ram et al., 2025; Walker, 2025b). After the bankruptcy was announced, State Attorneys General and privacy advocates issued urgent public warnings advising customers to delete their genetic data—and ask that the company destroy their biological samples—due to the risk of this sensitive, immutable information being sold as a corporate asset in the bankruptcy proceedings. In the following days, 23andMe received a deluge of inquiries and data deletion requests, crashing its consumer portal multiple times (Muoio, 2025). In the months that followed, nearly 2 million users, which represents about 15% of the company’s customer base, formally requested the deletion of their sensitive genetic data.

As legal commentators observed, neither federal privacy law nor existing research regulations clearly prevent the sale or transfer of genetic data during bankruptcy (Ram et al., 2025; Walker, 2025b). This legal ambiguity prompted a great deal of policy attention, including a US bill that would prohibit the transfer of identifiable genetic data during bankruptcy or corporate restructuring. Policy analysts also raised national-security concerns, arguing that bankruptcy could allow sensitive genomic datasets to be acquired by foreign entities in ways that circumvent existing regulatory oversight (Branch, 2025).

As prior studies have shown, experiences with DTC genetic testing influence public attitudes toward medical research participation, data sharing, and trust in genomic science (Boyce et al., 2019; Abitbol et al., 2022). The breach and bankruptcy amplified long-standing concerns about consent durability, data ownership, and commercial stewardship—not only for 23andMe’s participants but also for potential contributors to academic and public biobanks. Indeed, several public biobank leaders reported renewed participant inquiries about whether their data could ever be sold, transferred, or exposed in similar ways (Muoio, 2025).

23andMe’s trajectory reveals broader governance gaps in legacy planning, consent, and public expectations across the biobanking ecosystem, as discussed in Section 4 and 5 of this paper. The following subsection (3.3) examines one additional case study that helps complete the picture of the broader biobanking vulnerabilities laid bare by volatile commercial infrastructures.

### 3.3 DNA marketplaces: possibilities and limits of biobanking incentivization

In recent years, a growing number of commercial genomics companies have adopted the so-called “DNA marketplace” model—paying participants for data access and subsequently aggregating data for third-party access. These companies offer participants compensation, such as cryptocurrency or even company ownership stakes, in exchange for access to their genetic data (Walker, 2025b). Rather than collecting data primarily for in-house research, these platforms are designed to facilitate ongoing circulation of genetic information across academic, pharmaceutical, and commercial partners (Ahmed and Shabani, 2019). In contrast to direct-to-consumer testing companies that emphasize individual reports, DNA marketplaces are explicitly oriented toward aggregating data for research purposes—allowing users to either upload data from DTC companies, or sometimes offering free in-house sequencing as the form of payment.

As in other segments, organizational instability has been a recurrent feature of this sector, complicating the governance expectations attached to these models. Several DNA marketplace companies have shifted business models, revised data access terms, or closed altogether in recent years. Nebula Genomics, co-founded in 2016 by the controversial geneticist George Church, initially positioned itself as a privacy-forward alternative to mainstream consumer genetics, offering free or subsidized whole-genome sequencing in exchange for participant data-sharing through a blockchain-secured platform. Early iterations relied on token-based incentives, allowing contributors to receive proprietary cryptocurrency that could be used for platform services or exchanged externally (Walker, 2025b).

Over time, Nebula moved away from token-based incentives and reoriented its model toward subscription-based direct-to-consumer sequencing and sponsored sequencing arrangements. In these programs, researchers or commercial partners fund sequencing for individuals who meet specified criteria, while participants receive their raw genomic data and reports at no cost, and sponsors gain access to the resulting datasets for research use. Despite the apparent generosity of these incentives, Nebula executives have acknowledged persistent difficulty generating sustained participation. As Church himself remarked, “Even at free, people do not perceive the value,” highlighting the limits of sequencing-as-incentive (Begley, 2018).

By early 2025, Nebula reported approximately two million users and had entered into global collaborations to reach DTC genomics markets and possible participant pools internationally, for example, a partnership with MenaDNA focused on Middle Eastern countries including Jordan, Oman, and Iraq (MENADNA and Nebula Genomics Announce Strategic Partnership 2024). Nebula was acquired 5 years after its founding, by ProPhase Labs in 2021, but little change to consumer interfaces made this ownership shift opaque to participants (ThrowAwayGenomics, 2025). Yet, in corporate filings and investor communications, ProPhase explicitly framed Nebula’s accumulated genomes as key corporate assets, describing the company as having “compiled extensive whole genome data from 120+ countries, revealing a hidden and valuable asset,” and later emphasizing that its collection of whole-genome sequenced data “holds significant value” beyond the structures of the business itself (ProPhase Labs, Inc, 2024; ProPhase Labs, Inc, 2025). But these filings also signaled strategic uncertainty, including a search for a new purchaser—underscoring how participant-contributed genomic data may be repeatedly revalued and repositioned through corporate restructuring.

In 2024, ProPhase Labs reorganized Nebula Genomics as an internal sequencing laboratory and launched a new consumer-facing brand, DNA Complete, built on Nebula’s infrastructure. During this transition, existing users reported that “lifetime” subscriptions were cancelled, accounts were migrated without reconsent (even for recurring payments), and data-access and pricing terms were revised, often with limited advance notice (ThrowAwayGenomics, 2025). Participants who had contributed genetic data under one set of expectations about platform stability and access thus experienced a redefinition of those terms through corporate restructuring. In late 2024, Nebula users launched a class-action lawsuit alleging that Nebula shared users’ genetic data with third-party technology companies without authorization, highlighting how instability and changing business arrangements can expose participants to risks that extend beyond those anticipated at the point of consent (Justia Law, 2025).

LunaDNA presents a contrasting but related example of the DNA Marketplace case. Founded in 2017 by former leaders of the sequencing firm Illumina and supported by Illumina Ventures, LunaDNA initially experimented with cryptocurrency-based rewards before pivoting to an equity-sharing model. Participants could “rent” their DNA to researchers in exchange for shares in the company—for example, 300 shares for contributing a whole genome to the platform, which the company’s preliminary offering circular valued at approximately $21 (U.S. Securities and Exchange Commission, 2018). In addition to financial incentives, the platform was designed to support ongoing participant engagement: contributors could specify the types of studies they were willing to participate in, opt in or out of re-contact, and exercise more granular control over downstream data use than is typical in one-time consent models (Koplin et al., 2022). LunaDNA was incorporated as a public benefit corporation, a for-profit legal form that requires firms to pursue an articulated public benefit in addition to profit. The company’s premise was that by granting participants equity stakes, contributors would share financially in any proceeds generated through the licensing of their genetic data (Walker, 2022).

Despite this structure, LunaDNA was unable to establish a sustainable revenue stream and announced its closure in early 2024, reporting that it had no remaining cash reserves and therefore could not issue payouts to its participant-shareholders (Grinstein, 2024). When the company shut down, its genetic and other health datasets were not sold or transferred to another entity. The PBC structure and offering claims had clarified that participant-contributed genetic data were not to be treated as transferrable corporate assets (Rare Daily, 2024). The case highlights possibilities as well as limits in alternative governance models for biobanking: although LunaDNA offered contributors equity as a way to align individual participation with collective value creation, the absence of payouts underscores how uncertain and contingent such benefits are in practice.

By framing data contribution as a transactional or investment-like activity, DNA marketplaces influence how individuals understand ownership, compensation, and control in genetic research (Ahmed and Shabani, 2019). Emerging research suggests that interactions with commercial genomics platforms affect broader attitudes toward data sharing and research participation, with implications for trust in public and nonprofit biobanking initiatives as well (Briscoe et al., 2020; Walshe et al., 2024). In this sense, DNA marketplaces contribute not only to the commercialization of genetic data, but also to evolving public imaginaries of what biobanking entails—and what contributors can reasonably expect from those who steward their genetic information. The following section draws lessons from across these cases that inform governance across the biobanking ecosystem.

## 4 Discussion

By examining genomics companies that collect, store, and distribute genetic data at scale, these cases provide a vantage point for reflecting on how biobanking practices are changing, and where existing governance frameworks may be strained. They make visible a set of governance tensions—around continuity, incentives, and data value—that are often less explicit in discussions of traditional biobanks but are nonetheless relevant to them. Reading these cases alongside the existing biobanking scholarship helps examine the broader health of biobanking systems, how developments in commercial genomics intersect with broader biobanking governance, and why addressing those intersections matters for the future of biobanking as a whole.

### 4.1 Legacy planning and the problem of institutional impermanence

Across the cases examined in this paper, a shared governance challenge emerges: institutions change, fail, or dissolve, while genetic data and biospecimens can be made to persist. In commercial genomics, closures, pivots, acquisitions, and bankruptcies are not exceptional events but routine features of venture-backed innovation. As the cases of 54gene, DNA marketplaces, and 23andMe illustrate, genomic datasets may retain scientific and commercial value long after the organizations that assembled them lose financial viability or operational capacity. Yet governance frameworks rarely treat institutional impermanence as a core design constraint.

These cases expose persistent uncertainties about what happens to genetic data and samples when organizations restructure or shut down. Questions about custodianship and the permissibility of sale or transfer often surface only at moments of crisis, rather than being addressed prospectively. When genetic resources are treated as salable assets, participants and contributing communities may have little clarity about how consent commitments will be honored once data are sold, transferred, or repurposed. The result is not only legal ambiguity, but also heightened concern about commoditization and the erosion of protections after organizational transition.

These governance gaps persist despite the proliferation of legal frameworks intended to protect genetic data privacy and restrict commodification of human biological materials. The sale of human tissue is outlawed in most countries globally; in some countries (such as South Africa, and China) such tissue regulations also cover “bodily materials,” including genetic samples derived from saliva (Gooden and Thaldar, 2022). The recently implemented EU Policy on Substances of Human Origin also prohibits financial gain from the sale of tissues and bodily fluids (Elias et al., 2024). However, these statutes have seen limited legal implementation and enforcement, which has left ongoing legal debate about how existing provisions should be interpreted (Gooden and Thaldar, 2022; Santamaría, 2017; Cuende et al., 2025).

In the name of national security and the sovereignty of national resources, numerous countries also subject cross-border transfers of human genetic material—and in some cases associated data—to governmental review or approval (Chen et al., 2025; McKibbin and Shabani, 2023; Sathar and Dhai, 2012). Comprehensive data protection statutes, such as the European Union’s General Data Protection Regulation, have also expanded globally in recent years, placing additional restrictions on cross-border transfer of “sensitive data”—including genetic information (Becker and Dove, 2026). The United Kingdom, Nigeria, Brazil, South Africa, and India have all passed analogous laws, but to date none have been interpreted as prohibiting the transfer of datasets during corporate acquisition, nor as requiring re-consent, as long as the acquiring entity maintains comparable purposes and protections (Hauck, 2019; Bag et al., 2025).

Governance challenges related to organizational closure and the transfer of genetic resources are not confined to commercial settings. As highlighted in the section above on “Aging Biobanks,” public and nonprofit biobanks also face rising costs, underutilization, shifting funding priorities, and eventual closure—even sometimes resulting in sale of biobank assets prior to closure as in the CIRM case. Legacy planning in this arena remains limited, uneven, and poorly communicated to participants (Matzke et al., 2016; Rao et al., 2019; Zawati et al., 2011; Stephens and Dimond, 2015). While recent scholarship has begun to call attention to biobank sustainability and closure planning, most biobanks still lack explicit policies governing long-term stewardship, data transfer, or participant notification in the event of institutional decline (Wosen, 2025; Tupasela and Stephens, 2013; Baláž et al., 2022). As a result, participants in ostensibly stable public biobanks may face governance uncertainties similar to those revealed more starkly in commercial contexts.

These parallels also caution against framing the problem as one of “commercialization” alone. Public and nonprofit biobanks have long faced sustainability pressures, including rising costs, underutilization, and institutional restructuring. Financial relationships and revenue strategies are therefore not new or unique to commercial genomics. The concern raised here is not the presence of financial logics, but the opacity of those arrangements and the absence of enforceable custodial safeguards when genetic resources are treated as transferable assets. Governance reform must address sustainability and transparency together, rather than positioning commercialization itself as the core risk.

Some established public biobanks have begun to articulate clearer legacy provisions. For example, UK Biobank’s participant materials state that if the resource were to close or reach the end of its “natural life,” stored biological samples would be destroyed (UK Biobank, 2009). This explicit destruction clause reflects an effort to anticipate institutional impermanence and provide prospective clarity regarding sample disposition. At the same time, such provisions raise further questions about whether destruction, transfer, or continued managed access best honors participant expectations—particularly where contributors may have joined with the hope that their samples would support long-term research in the public interest.

These examples highlight the importance of legacy planning, but also suggest benefits from treating it as a shared governance design problem across the biobanking ecosystem. Rather than treating closures as exceptional failures or uniquely commercial risks, this perspective recognizes the realities of institutional turnover as a feature of contemporary biobanking infrastructures. It also creates a basis for aligning public, nonprofit, and commercial actors around shared responsibilities for continuity, transparency, and participant protection. Seen in this light, companies-as-biobanks do not introduce entirely new problems, but instead surface—at accelerated speed—the governance gaps that increasingly affect biobanking as a whole. The following subsection focuses on an additional gap in this vein: the question of participant incentivization.

### 4.2 Incentives, returns, and participant expectations

DNA marketplaces have aimed to introduce new participation and incentivization models, and as a result they function as experimental sites for efforts that traditional biobanks are increasingly being asked to consider (Spector-Bagdady et al., 2018; Lin et al., 2024; Tigard, 2019). Platforms such as LunaDNA and Nebula have explicitly tested alternatives to altruistic participation, including equity-based profit sharing, free or subsidized sequencing, and enhanced access to personal genetic information. These models reflect growing pressure across biobanking to articulate clearer and more tangible benefits for participants, often to aid in recruitment.

Debates over incentivization have occupied a growing place in biobanking and ELSI scholarship (Slobogin et al., 2025; Majchrowska et al., 2024; Sanderson et al., 2017; Guo et al., 2023; Beltrame, 2019; Sýkora et al., 2013). Critics have argued that financial or quasi-financial incentives risk commoditizing biological materials and research relationships, undermining norms of solidarity and reframing participation as market exchange rather than collective contribution (Tigard, 2019; Prainsack and Buyx, 2013). From this perspective, participation grounded in solidarity—rather than payment—is seen as essential to maintaining public-oriented biobanking infrastructures, with some commentators even proposing compulsory genomic databases as a way to avoid inequities generated by the combination of market-based recruitment and forensic databases (Hazel et al., 2018). These debates underscore that incentive design is not merely a pragmatic question of recruitment, but a normative choice about how biobanking relationships are structured (Saha and Hurlbut, 2011; Solberg and Steinsbekk, 2015; Stranger, 2009; Goodwin, 2013; Karavas, 2020).

Existing literature on the return of individual research results suggests that non-monetary benefits—such as clinically relevant or personally meaningful genetic findings—may be a way to support participation without reframing research relationships as commercial exchange, although there is disagreement as to whether such structures do indeed bypass the problems of commoditization of research relationships (Lekstutiene et al., 2021). Across ELSI scholarship, there has been growing convergence around the view that results should be returned when findings are analytically valid, clinically significant, and actionable, and that participants should be able to consent to the return of such information—while also attending to questions of feasibility, resource constraints, and the potential for harm or misunderstanding (Gibson et al., 2019; Lin et al., 2019; Viberg et al., 2014). In biobanking contexts, this literature has further emphasized that responsibilities for return of results are distributed unevenly across actors, with different obligations attaching to the point of collection, to biobanks as intermediaries, and to downstream secondary users (Wolf et al., 2020).

Within this context, DNA marketplaces are shaping public experiences regarding transparency and participant engagement. At the same time, these platforms expose the limits of incentive structures that move toward the language of assets, investment, ownership, and profit. When companies or other biobanking entities emphasize speculative financial returns for participants, those participants may develop expectations that are difficult to satisfy—especially when companies fail to generate revenue, change business models, or cease operations altogether—highlighting the fragility of incentive regimes grounded primarily in market logics.

Yet, DNA marketplace experiments suggest that new incentive models are neither inherently misguided nor easily dismissed. Offering ownership stakes or profit-sharing can be appealing precisely because it acknowledges a reality that many participants already perceive: genomic data are monetized, whether or not contributors share in that value (Jabloner and Walker, 2023; Briscoe et al., 2020). In this sense, models that recognize participants as stakeholders may be more honest about contemporary data economies than frameworks that insist on altruism while commercial actors capture downstream benefits. At the same time, these cases demonstrate that symbolic ownership or speculative profit-sharing risk exploiting users by overpromising (Walker, 2025b). While company executives have often earned hefty paychecks using participant genetic data as a company’s financial motor, payouts have failed to materialize. But robust, transparent, and realistically attainable payout structures could potentially alter those relationships in favor of more substantive profit-sharing.

For biobanking governance, the lesson is not that incentives should be abandoned, but that they must be coupled with robust protections, realistic benefit structures, and clear limits on commodification. The example of Nebula Genomics further illustrates that even seemingly generous incentives—such as free whole-genome sequencing—do not automatically translate into sustained participation. This suggests that incentives must be closely aligned with participants’ concrete needs and priorities, such as clinically meaningful information, while also guarding against forms of exploitation that arise when health needs are leveraged to secure data contribution. These tensions cannot be resolved through consent processes alone (Teare et al., 2021; Dankar et al., 2019; Wiertz and Boldt, 2022). Instead, they point to the need for governance frameworks that combine robust benefit-sharing, limits on commodification, and durable protections—so that incentives promote solidarity and reciprocity without shifting undue risk onto participants. The next subsection turns to these questions in the context of genomics diversification efforts.

### 4.3 Assetization, diversity, and shifting ethical obligations

Across contemporary biobanking, genomic diversity has increasingly been framed not only as a scientific necessity but as a source of economic value (Jabloner and Walker, 2023; Petteway, 2023). In commercial genomics, datasets derived from populations historically underrepresented in research—particularly African, diasporic, and global indigenous populations—are often positioned as both ethically corrective and commercially strategic (Lee et al., 2025; Jackson et al., 2019). This dual framing is evident in claims that expanding representation will improve precision medicine while also generating valuable inputs for pharmaceutical, diagnostic, and other analytics development. As critics have noted, however, the financial gains associated with expanding genetic datasets and accessing new markets can eclipse justice-based rationales, allowing companies to leverage the moral language of diversity while aligning inclusion primarily with investor and growth imperatives (Tsosie et al., 2021; Tupasela et al., 2015).

Assetization reshapes what inclusion means in practice. Rather than addressing historical exclusion primarily through public investment, institutional accountability, or community benefit, diversity is increasingly operationalized as a resource to be assembled, owned, and leveraged (Kishore, 2006; Smith, 2008; Laurier Decoteau, 2013). This dynamic echoes longstanding analyses of hypervisibility and invisibility in science and technology: marginalized populations remain underrepresented in the benefits of biomedical research, yet become highly visible when their data are newly valuable (Noble, 2013; Braun et al., 2023; Bradley, 2025). Participation is encouraged at moments of extraction, while pathways to tangible benefit remain diffuse, deferred, or undefined.

Much of the ethical justification for these models relies on what might be called a “trickle-down science” narrative: the idea that increasing diversity in genomic datasets will eventually yield more equitable health outcomes (Carter and Maricque, 2025). Yet these claims often lack concrete mechanisms linking data contribution to present-day benefits for participants or their communities. Promises of future inclusion in research or eventual therapeutic relevance do little to address immediate questions of health inequity and the benefits of research contribution for members of marginalized groups—particularly when data are mobilized within infrastructures subject to volatility, restructuring, or sale.

In the US, observers often cite the Henrietta Lacks case as an example of how value has historically been extracted from biospecimens of members of marginalized groups, without financial or healthcare benefit accruing to them or their families (Baptiste et al., 2022; Nisbet and Fahy, 2013; Menikoff, 2025). The absence of consent is a central ethical failure in the Henrietta Lacks case, reflecting broader histories of racialized exploitation in biomedical research. Many observers today find it particularly reprehensible that Ms. Lacks herself died without adequate care, and that her descendants and broader community have long lacked reliable access to healthcare, even as HeLa cells became foundational to modern biomedicine (Dimaano and Spigner, 2017).

But the ethical lessons of the HeLa case extend beyond questions of consent or profit-sharing. The fact that HeLa cells generated immense scientific and economic value is not the reason that Ms. Lacks, her descendants, or her community are owed healthcare and opportunity; these are rights owed as a matter of basic justice and human dignity (Walker et al., 2021; Creary, 2021; Eleftheriadis, 2012). Yet the case reveals how biomedical systems have drawn value from the bodies and lives of marginalized populations while failing to ensure that those same populations have access to the social and health benefits those systems help produce. For contemporary biobanking, this underscores that expanding “diversity” in genomic datasets cannot be treated as an ethical endpoint in itself. Instead, biobank governance must grapple with questions of reparative obligation, including how institutions that rely on the biological contributions of historically marginalized groups can promote durable protections, accountability, and health equity in the present—not only through speculative future benefits of research (Soled et al., 2021; Harman et al., 2021; Agozino, 2021).

Seen through this lens, the assetization of genomic diversity raises questions beyond individual consent or compensation. It challenges biobanking institutions—public and commercial alike—to articulate what is owed when participation is actively solicited from populations long excluded from biomedical benefit, and when inclusion itself becomes a driver of value creation. Addressing these questions requires moving beyond abstract appeals to diversity toward governance arrangements that foreground accountable stewardship, present-day benefit, and ecosystem-wide responsibility. The following section synthesizes lessons from these cases to propose such governance recommendations for the broader biobanking ecosystem.

## 5 Policy takeaways

### 5.1 Governing biobanking across commercial, public, and platform contexts

The case studies presented here have illustrated how today’s genomics companies—and especially those that function as *de facto* biobanks—surface governance problems for biobanking that extend well beyond any single organizational form. Issues of continuity, participant protection, incentive design, and the treatment of genetic data as salable resources recur across commercial and public settings alike, even as they take on distinct forms depending on institutional context. What these cases make visible is not a simple contrast between “good” public biobanks and “problematic” commercial ones, but a shared set of pressures arising from how genetic data are now collected, stored, circulated, and valued over time.

Taken together, these findings point to the need for governance approaches that operate at the level of the biobanking ecosystem as a whole—one composed of public and nonprofit repositories, academic–industry partnerships, genomics companies, and data platforms whose activities are increasingly interdependent. The following section builds on this analysis to outline governance directions suited to this contemporary assemblage, focusing on institutional arrangements that can support continuity, stewardship, and participant protection across shifting organizational and technical landscapes.

### 5.2 From choice to structural protection

Biobanking governance has relied heavily on informed consent as a way to manage ethical and legal risks—particularly risks related to privacy, secondary use, and misuse—while also signaling respect for participant preferences and legitimating downstream uses as authorized by people that contributed data and samples (Alkhatib et al., 2024; Akyüz et al., 2021; Coppola et al., 2019). Extensive debates have examined the legitimacy of consent mechanisms that allow researchers to use samples for a broad range of studies unspecified to participants, the appropriateness of consent for secondary and unforeseen uses, and the kinds of research—commercial, sensitive, or socially contested—that should or should not be permitted within biobanking infrastructures (Steinsbekk et al., 2013; Mikkelsen et al., 2019; Manson, 2019; Caulfield and Kaye, 2009; Ploug and Holm, 2020; Manson, 2019). In response to well-documented limits of one-time informed consent for addressing this range of issues, scholars and practitioners have developed important innovations, including dynamic consent platforms and decision aids designed to support ongoing participant engagement and choice in data use (Thiel et al., 2014; Zhou et al., 2019; Prictor et al., 2018).

These debates have highlighted not only cognitive limits of participant understanding in biomedical research, but also the structural conditions that make comprehension difficult in practice: not only the technical complexity of genomics and related studies, but also the long time horizons of biobanking, and the involvement of multiple institutions and downstream users whose identities and interests may change (Walker, 2025b). From this perspective, the central concern is less whether participants can be made to understand everything in advance, and more whether governance arrangements can reliably protect participants when understanding is partial, uneven, or necessarily provisional.

The cases examined in this paper make these governance tensions visible by revealing how approaches grounded primarily in informed consent are strained by organizational change and financial pressure. Commercial genomics companies operate at the intersection of research, consumer markets, and data economies, where participation is framed through branding, promised benefits, and public-facing narratives as much as through formal consent. Their prominence and volatility highlight a central challenge for biobanking governance: the widening gap between expectations formed at the point of participation and the realities of long-term data stewardship.

These dynamics are not unique to commercial genomics. As public and nonprofit biobanks age, face financial pressure, or undergo restructuring, similar challenges arise around continuity, responsibility, and the durability of protections over time. Taken together, these cases underscore the need for governance approaches that do not rely primarily on participant understanding, but are designed instead to be sustained across institutional change, and evolving research and commercial landscapes. The sections that follow examine what these safeguards might entail in practice.

### 5.3 Stewardship across institutional change: limits on assetization

An emerging biobanking literature has begun to draw attention to questions of closure, succession, and legacy planning, particularly as early biobanks age and as funding landscapes have shifted away from long-term infrastructure support toward shorter grant cycles, project-based financing, and public–private cost-sharing arrangements (Zawati et al., 2011; Caulfield et al., 2014; Matzke et al., 2016). These dynamics mean that even biobanks founded with explicit public-interest mandates are not immune to financial strain, restructuring, or closure. Genomics companies, by contrast, are structurally exposed to market volatility, investor timelines, and strategic pivots, making organizational change a routine feature. Because these companies operate in highly visible, public-facing ways, moments of closure, restructuring, or asset sale have immediate implications for public confidence that genetic samples and data will be protected, stewarded responsibly, and not treated as liquid assets.

Across public and commercial contexts, these moments of institutional transition are also moments when genetic data and biospecimens are most likely to be treated as transferable assets. As we have seen, treating biobank resources as salable property during such moments can create powerful incentives that may conflict with participant expectations and ethical commitments. Allegations surrounding 54gene, for example, suggest how investors may be motivated to push organizations toward insolvency precisely in order to liquidate valuable collections of samples and data. Importantly, this risk is not confined to commercial genomics. The recent closure of the California Institute for Regenerative Medicine’s stem cell biobank illustrates how even publicly funded infrastructures can come to treat biological materials as salable assets, especially when an organization is under financial pressure—raising parallel concerns about access, stewardship, and public accountability. While the CIRM case involved the sale of individual cell lines rather than an entire database, it underscores that full-resource sale at closure is not unimaginable even in public biobanking.

At the same time, participant expectations differ meaningfully across biobanking contexts, with important implications for governance. In the case of companies such as 54gene, where the company frames participation explicitly around research contribution, expectations may more closely resemble those associated with traditional research biobanks than consumer-facing genetic testing services. Although public globally have limited awareness of biobanks, research across national contexts has shown that participation is often motivated by commitments to scientific contribution and health solidarity, as well as expectations that samples will continue to be used for research that benefits broader publics (Mackenzie, 2015; Akinyemi et al., 2018; Igbe and Adebamowo, 2012). Emerging empirical evidence suggests that many contributors in such contexts prefer that, if an institution can no longer operate, their samples be transferred to a public or nonprofit repository and continue to be used to advance the kinds of research that originally motivated their contribution—and not be sold, destroyed, or repurposed primarily for profit-oriented activities (Wosen, 2025; Broekstra et al., 2022; Caulfield et al., 2014). These expectations provide concrete guidance for ethically defensible approaches to biobank closure and transition.

The highly visible case of 23andMe has played a central role in catalyzing legal and policy attention to the treatment of genetic data during corporate restructuring and closure. In the United States, for example, the proposed Don’t Sell My DNA Act would prohibit the transfer of identifiable genetic data during bankruptcy or similar proceedings. These are important protections. Yet, in the context of companies like 54gene or other research-oriented biobanking contexts—whether housed in public institutions or commercial entities—many contributors reasonably expect that their samples and data will continue to support research rather than being abandoned, destroyed, or redirected toward unrelated commercial purposes (Wosen, 2025). In addition to prohibitions on sale, there may therefore be strong moral grounds for legally requiring public or nonprofit stewardship as the default endpoint in cases of closure or restructuring across the biobanking ecosystem—including consumer-services-as-biobanks, research-oriented genomics firms, and traditional public or nonprofit biobanks. At the same time, this option requires careful governance, especially given the widespread use of public–private partnerships and managed access arrangements in contemporary biobanking that strengthen profit motives as well as participant concerns. Protecting contributors’ expectations in moments of transition requires explicit limits on assetization alongside enforceable custodial obligations that prioritize continuity of meaningful public benefit.

Such protections cannot rely on biobank self-governance alone, particularly because many companies-as-biobanks fall outside traditional oversight frameworks in this arena. Instead, limits on assetization and requirements for continuity planning could be embedded in institutional authorization and oversight mechanisms, including conditions attached to public or philanthropic funding, regulatory approval for large-scale data collection or transfer, research ethics oversight, and—where genomics companies operate as consumer services—data protection and consumer protection law. Making these safeguards explicit at points of formation, acquisition, restructuring, or closure is essential to ensuring that custodianship obligations persist even as organizations change form.

Taken together, the cases examined in this paper highlight the need for baseline continuity requirements across the biobanking ecosystem. Legacy planning should be required of any organization that undertakes biobanking activities at scale—that is, the long-term collection, storage, governance, and third-party sharing of genetic data or biospecimens—regardless of whether that organization identifies itself as a biobank. This includes public and nonprofit biobanks, hybrid public–private initiatives, and genomics companies that function as *de facto* custodians of genetic resources. Rather than treating continuity as an expectation, such requirements should be integrated into routine governance processes tied to authorization, funding, and expansion.

Framing continuity and legacy planning as an ecosystem-wide obligation helps normalize institutional transition as a foreseeable governance challenge. It also aligns governance more closely with participant expectations that stewardship obligations and contribution to the public good persist long-term, shifting protection away from assumptions of institutional permanence toward enforceable commitments that can withstand organizational change across diverse biobanking settings. The next subsection turns to questions of incentivization that shape these participant expectations.

### 5.4 Companies-cum-biobanks as experimental sites for benefit-sharing and incentives

With increasing recruitment competition created by the proliferation of biobanks and large genomics research efforts, as well as growing efforts to recruit more ancestrally diverse populations, biobank recruitment has become particularly challenging in recent years (Goddard et al., 2009; Amin et al., 2021; Boutin et al., 2022). Debates have increasingly turned to questions of incentivization, and whether monetary or other incentives ought to be offered to increase participation (Tigard, 2019; Bailey et al., 2021; Meitern and Hansson, 2023; Lekstutiene et al., 2021; Briscoe et al., 2020). At the same time, public understandings of the economic value of genetic data have grown markedly (Briscoe et al., 2020). Alongside broader efforts to involve participants and publics in shaping research agendas to align them with the public interest, these evolutions have prompted a search for alternative research participation models (Lekstutiene et al., 2021; Tigard, 2019; Briscoe et al., 2020).

DNA marketplace platforms have emerged within this landscape as experimental sites for testing alternative participation models, including equity-based profit-sharing, free or subsidized genome sequencing, enhanced access to personal genetic information, and greater participant control over data use. These experiments are instructive not because they offer ready-made solutions, but because they make visible the trade-offs involved in aligning participation, benefit, and governance in settings where genetic data clearly generate value.

Growing concern that biomedical research often extracts value from participants’ contributions without delivering meaningful benefits has driven increased attention to benefit-sharing in bioethics and ELSI scholarship (Ganguli-Mitra, 2008; Simm, 2005; Hayden, 2007; Cartney et al., 2024). This literature has developed alongside debates about incentives and undue inducement, although the two bodies of work have not always been in close dialogue—despite the fact that benefit-sharing can be understood as a response to longstanding concerns about the possibly coercive effects of incentives. At the same time, critics have argued that concerns about undue inducement have sometimes been overstated; we ought not worry about incentives being too high in today’s context of substantial downstream financial and scientific value being produced while even the highest payments do not come close to allowing contributors to meaningfully share in these financial benefits (Emanuel, 2005; Ballantyne, 2008; Emanuel, 2004).

The literatures on incentivization and benefit-sharing differ in their framings. Incentives are typically understood as payments or rewards offered to motivate participation; benefit-sharing, by contrast, focuses on how the value generated through research—including financial value—is distributed over time to contributors and communities as a matter of fairness, reciprocity, and justice (Lekstutiene et al., 2021; Tigard, 2019; Simm, 2005; Schroeder, 2007). Proposed benefit-sharing approaches have included the return of individual research results where clinically valid and appropriate; access to health-relevant knowledge, diagnostics, or technologies developed using contributed data; reinvestment in local or regional health infrastructure; and sustained support for research capacity in settings whose populations are disproportionately drawn upon for data (Schroeder and Gefenas, 2012; Matsuyama et al., 2023).

The justice theories discussed earlier in this paper help clarify why these distinctions matter ethically. Reparative justice directs attention to how contemporary biomedical research builds on long histories of unequal contribution and benefit (Harman et al., 2021; Agozino, 2021; Soled et al., 2021; Danieli, 2009). The work of scientific institutions—particularly those based in Europe and North America—have been made possible not only by biological materials and data, but also by forms of labor, extraction, and social inequality that have disproportionately burdened marginalized populations while concentrating resources elsewhere (Agozino, 2021; Anderson, 2006; Benjamin, 2014; Seth, 2009; Hu, 2025; Law and Lin, 2017; TallBear, 2013). From this perspective, researchers and institutions have a general obligation to pursue health equity. But these obligations intensify when research and innovation draw directly on the biological materials, data, or participation of populations that have been historically excluded from the benefits of biomedical advance (Epstein, 2024; Sharp and Foster, 2007; Orth and Schicktanz, 2016). Framed this way, benefit-sharing is not a reward for participation, but a mechanism for meeting heightened obligations that arise when value is extracted from already disadvantaged groups.

The DNA marketplace cases examined here illustrate both the appeal and the limits of market-oriented incentive models. Equity stakes or profit-sharing arrangements can be attractive precisely because they acknowledge an underlying reality: genetic data are monetized, whether or not contributors share in that value. In this sense, cooperative or ownership-based models may be more transparent than frameworks that rely exclusively on altruism while allowing value extraction elsewhere. At the same time, these cases show that speculative or symbolic financial returns are fragile and often fail to deliver meaningful benefit, particularly when companies pivot, restructure, or close.

Taken together, these experiences suggest that more durable participation models do not treat contribution as an investment opportunity with uncertain returns, but instead tie benefits to the rules and institutions that govern how biobanks operate over time. For biobanking efforts—whether public, nonprofit, or commercial—this means specifying, in advance, concrete ways for contributors to benefit from downstream research use: through access to findings, access to resulting tests or therapies, reinvestment in health infrastructure, or other defined public-interest commitments. This way, benefit-sharing is not an optional add-on or goodwill gesture, but part of the governance architecture that determines how value generated from genetic data is distributed.

The most ethically robust forms of benefit-sharing are therefore those that address downstream access: affordable availability of diagnostics, tests, or therapies developed using contributed data; preferential or guaranteed access for contributing populations; and reinvestment of revenues into health systems that serve those populations. For contributors from historically underrepresented or marginalized groups, such measures directly confront a recurring pattern in which inclusion in research generates value for research institutions while existing health inequities persist. Biomedical researchers and institutions hold general obligations to health equity and to the public interest—as members of society but especially as recipients of public funding (Keestra, 2021; Caulfield and Ogbogu, 2015; Contreras, 2021; Yamey, 2008). But they also bear differentiated obligations to those with whom they have direct and ongoing relationships—namely, the individuals and communities who contribute samples and data and who assume risks in the present (Ewuoso et al., 2022). Companies-as-biobanks make these differentiated obligations harder to ignore, precisely because they render value extraction visible. Meeting those obligations requires governance arrangements that translate participation into tangible improvements in health knowledge, access, and care, rather than relying on abstract future benefit or individual consent alone (Yamey, 2008). The following section turns to governance approaches at the ecosystem level that can support these goals.

### 5.5 Biobanks and ecosystem-level governance

Traditional public and nonprofit biobanks occupy a distinctive position within the contemporary biobanking ecosystem. They retain comparatively high levels of public trust, long-standing ethical and regulatory expertise, and a direct stake in the sustainability of shared research infrastructures. As commercial genomics companies increasingly perform biobank-like functions—and shape public expectations about data stewardship—governance failures in one part of the ecosystem now reverberate across others.

For this reason, traditional biobanks can be thought to have responsibilities, or at least interests, in ecosystem governance that extend beyond their own repositories. Policy literatures in fields such as financial regulation, data protection and privacy, and environmental management all suggest a role for such public interest actors in the regulation of multi-institutional ecosystems (Yeung and Bygrave, 2022; McIntyre, 2018; Scherer and Voegtlin, 2020). Models from these literatures suggest that traditional biobanks’ regulatory engagement is most appropriate at moments in the biobanking landscape where participant protections are most vulnerable and where existing oversight is fragmented: large-scale data aggregation, secondary data access, corporate restructuring or acquisition, and biobank closure or transition. In these arenas, companies-as-biobanks currently fall outside established biobanking governance frameworks, yet exercise *de facto* custodial power over genetic resources.

Traditional biobanks are well positioned to help convene and shape standards that apply across institutional forms in this ecosystem. Such processes could be coordinated through existing international and national bodies with experience in biobank governance and research ethics, such as international genomics consortia. Policy literatures also suggest that, to have impact and legitimacy, these standards should be developed through multi-stakeholder processes that include participant representatives, public-interest organizations, regulators, and commercial actors, with the explicit aim of producing baseline, enforceable expectations rather than voluntary best practices alone (Fransen, 2012; Scherer and Voegtlin, 2020; Hemmati et al., 2002).

Finally, traditional biobanks have an important role to play in resisting siloed governance. When ethical oversight, commercialization, data protection, and consumer regulation are treated as separate domains, gaps emerge that undermine stewardship and public trust. By advocating for integrated governance approaches—across research regulation, data protection law, funding conditions, and consumer protection—public-interest biobanks can help ensure that norms developed for biobanking continue to shape the governance of genetic resources, even as organizational forms and data economies evolve.

## 6 Next steps for leaders in industry, biobanking, and policy

### 6.1 For policymakers and regulators

#### 6.1.1 A package of governance levers

Different jurisdictions will require different tools depending on existing legal infrastructure, political will, and regulatory capacity. Rather than a single reform model, policymakers can draw on a package of possible levers:

- Clarify asset-transfer rules. Through legislation, regulatory guidance, or judicial interpretation, specify whether genetic datasets and biospecimens may be transferred during acquisition, restructuring, or insolvency—and under what conditions.
- Adopt default public stewardship safeguards. In cases of bankruptcy, require that biobank-scale genetic resources transfer to a qualified public or nonprofit custodian unless an alternative arrangement receives independent approval.
- Leverage existing oversight points. Data protection authorities, health research regulators, and public funders can attach continuity-planning requirements to accreditation, ethics approval, or funding conditions without awaiting comprehensive statutory reform.

### 6.2 For leaders in industry

#### 6.2.1 Transparency and participant-facing safeguards

- Communicate clearly how genetic data and biospecimens generate revenue. This should be included in participant-facing materials.
- Offer prospective opt-in choices regarding asset transfer. Consent processes can include advance choices regarding transfer in the event of acquisition or sale, rather than treating such decisions as *post hoc* disclosures.
- Align ethics with recruitment and retention strategy. An emerging literature suggests that visible commitments to stewardship and transparency can function as a competitive advantage in attracting both talent and participants (Walker, 2025b).

### 6.3 For public and nonprofit biobank leadership

#### 6.3.1 Ecosystem engagement and prospective clarity

- Publish explicit legacy and closure plans. Address custodial succession, sample destruction, transfer, and participant notification, before crisis moments.
- Participate in policy deliberations about assetization. Public biobanks are not insulated from financial pressures and should help shape ecosystem-wide standards rather than treating commercial actors as external.
- Communicate financial realities transparently. Acknowledge funding pressures and public–private partnerships to avoid reinforcing unrealistic assumptions of institutional permanence.

#### 6.3.2 Barriers and pathways forward

Reform faces predictable constraints: fragmented regulatory authority; reluctance to constrain high-growth biotechnology sectors; funding pressures in public biobanks; and limited political appetite for technical insolvency reform. Progress may depend on institutional champions—groups or individuals within data protection authorities, parliamentary committees, major funders, and professional consortia. Incremental strategies—regulatory guidance, funding conditions, voluntary standards, and visible transparency commitments—may build momentum toward more durable safeguards.

## 7 Conclusion

Genomics companies now play a central role in the collection, circulation, and stewardship of genetic data and biospecimens, even when they are not formally recognized or governed as biobanks. Examining these companies as *de facto* biobanking actors reveals governance challenges that are not unique to commercial genomics, but increasingly characteristic of the biobanking ecosystem as a whole. Across public, nonprofit, and commercial settings, biobanking now unfolds amid institutional volatility, financial pressure, and expanding data mobility—conditions that strain governance frameworks premised on stability, informed consent, and institutional continuity.

By situating companies-as-biobanks within the longer history of biobanking, this paper shows how issues of continuity, custodianship, assetization, and benefit-sharing are coming to the fore across the ecosystem. The case studies illustrate that governance failures are not simply the result of bad actors or weak consent processes, but reflect structural mismatches between contemporary genomic data economies and legacy models of research oversight. In particular, moments of restructuring, acquisition, and closure expose the limits of consent-based protections and highlight the need for enforceable obligations that persist across institutional change.

Taken together, these findings underscore the importance of ecosystem-level governance approaches that cut across institutional forms. Protections for participants and contributors cannot hinge on whether an organization self-identifies as a biobank, nor on assumptions of organizational permanence. Instead, they require shared standards for continuity planning, limits on the treatment of genetic resources as liquid assets, and benefit-sharing frameworks that translate participation into durable public and health benefits—especially where research draws disproportionately on populations that have historically borne the burdens of biomedical innovation without equitable return.

Public and nonprofit biobanks, with their credibility, expertise, and public-interest mandates, are well positioned to play a coordinating role in this shifting landscape. Here, engaging with commercial actors, regulators, and policymakers to help shape governance arrangements is not simply a matter of accommodating commercial genomics, but of safeguarding the long-term legitimacy, trustworthiness, and social value of biobanking itself.

## Data Availability

The original contributions presented in the study are included in the article/supplementary material, further inquiries can be directed to the corresponding author.
